# Reshaping relational social capital in the digital age: how digital tool usage influences the dual trust outcomes

**DOI:** 10.3389/fpsyg.2026.1759562

**Published:** 2026-05-15

**Authors:** Yanze Wang, Xinran Lyu

**Affiliations:** 1 School of Statistics and Mathematics, Central University of Finance and Economics, Beijing, China; 2 Chinese Academy of Finance and Development, Central University of Finance and Economics, Beijing, China

**Keywords:** communication quality, digital surveillance, digital tool usage, feeling trusted, psychological safety, technostress, trusting others

## Abstract

Digital technologies have fundamentally transformed workplace interactions, yet our understanding of how these tools reshape relational social capital, specifically trust dynamics, remains incomplete. Drawing on signaling theory and social exchange theory, this study examines how digital tool usage intensity influences two distinct dimensions of social capital: employees’ feeling of being trusted and their trust in others. Using two-wave time-lagged survey data from 428 employees in Chinese organizations, we find that digital tools function as a double-edged sword: they foster social capital through communication quality and psychological safety, while introducing “social risks” via perceived digital surveillance and technostress. Our findings advance social capital theory in the digital age by distinguishing feeling trusted and trusting others as parallel yet distinct outcomes, and by demonstrating that technology implementation choices carry powerful symbolic meanings that can either build or erode the organizational social fabric.

## 1 Introduction

Digital technologies have fundamentally transformed how organizations operate. From instant messaging platforms and collaborative software to virtual reality (VR) interfaces and IoT-based operational dashboards, these tools have become deeply embedded in contemporary work life (Ou and Davison, 2011; Slater and Sanchez-Vives, 2016; Zhang et al., 2025). Foundational IoT research conceptualizes IoT as an interconnected infrastructure of sensing, monitoring, and information-sharing technologies that increasingly underpins organizational processes and enterprise applications (Atzori et al., 2010; Lee and Lee, 2015; Westergren et al., 2024). What began as supplementary communication channels has evolved into pervasive digital environments that structure how employees interact, coordinate, and make sense of organizational reality (Zhou et al., 2025). While substantial research has documented the effects of digital technologies on operational efficiency and productivity (Amin et al., 2024; Ding et al., 2024), a critical dimension remains underexplored: how the intensification of digital tool usage fundamentally reshapes interpersonal trust dynamics in the workplace.

Trust serves as a foundational element of organizational effectiveness, enabling cooperation, reducing transaction costs, and catalyzing innovation (Ring and de Ven, 1994). The management literature distinguishes between two complementary yet distinct dimensions of workplace trust: trusting others, which reflects an employee’s willingness to accept vulnerability in relation to colleagues or supervisors, and feeling trusted, which captures an employee’s perception that others place confidence and reliance in them. Despite the centrality of trust to organizational functioning, our understanding of how digital work environments influence these trust dynamics remains theoretically incomplete.

Existing research on trust in digital contexts suffers from two significant limitations. First, prior studies have predominantly focused on discrete work arrangements such as virtual teams or remote work settings (Mutha and Srivastava, 2023), treating digital technology as a binary condition rather than examining how varying intensities of digital tool usage affect employees across different work configurations. This approach overlooks how the depth and pervasiveness of digital integration into daily workflows, whether through VR-enabled training, real-time IoT monitoring, or algorithmic task allocation, shapes employee psychological states regardless of physical location. Research on immersive media further suggests that VR differs from conventional communication tools because its affordances of vividness and interactivity heighten telepresence and social presence in digitally mediated collaboration (Steuer, 1992; Cummings and Bailenson, 2016; Sivunen and Nordbäck, 2015). Meanwhile, digital coordination systems increasingly incorporate algorithmic forms of direction, evaluation, and discipline rather than merely neutral information exchange (Kellogg et al., 2020; Leonardi, 2020). Second, and more fundamentally, the literature presents conflicting theoretical accounts of how digital tools influence trust. One perspective emphasizes that digital platforms enhance transparency, facilitate information sharing, and create traceable communication records, thereby strengthening trust relationships (Centobelli et al., 2022). A contrasting view highlights how these same affordances enable continuous monitoring and surveillance, evoking panopticon-like dynamics that undermine employees’ sense of being trusted and their willingness to extend trust to others (Zuboff, 1988; Sewell and Wilkinson, 1992; Kellogg et al., 2020). This theoretical divide suggests that the relationship between digital tool usage intensity and workplace trust is neither straightforward nor unidirectional. Rather, it likely operates through multiple competing psychological mechanisms that can simultaneously enhance and undermine trust. Understanding these mechanisms and the boundary conditions under which they operate has important implications for both theory and practice.

This study opens the black box of how digital tool usage differentially shapes the reciprocal dynamics between trusting others and feeling trusted. We propose that digital tools operate as a double-edged sword, simultaneously conveying signals of empowerment and control. On one hand, intensive digital tool usage can enhance communication quality and strengthen social connections, reduce information asymmetries, and cultivate psychological safety. These mechanisms, in turn, foster both the willingness to trust others and the perception of being trusted. On the other hand, the pervasive digital traces that employees leave through their tool usage raise concerns about algorithmic management and continuous oversight. When employees perceive digital tools primarily as surveillance instruments or experience them as sources of technostress, the positive trust-building effects of technology may be suppressed or even reversed. Under these conditions, employees may question whether the organization genuinely trusts them, leading them to withhold their own trust in response.

To address this theoretical puzzle, we develop an integrated framework drawing on signaling theory and social exchange theory. We examine how digital tool usage, encompassing collaborative platforms, VR, and Internet of Things devices, influences both feeling trusted and trusting others through dual mediating pathways: communication quality and psychological safety. Critically, we introduce perceived digital surveillance and technostress as boundary conditions that shape whether digital tool usage strengthens or undermines these trust dynamics. Through survey data collected from employees in digital sectors, we capture the complex interplay between technology adoption and trust formation in contemporary organizations.

This study makes three primary contributions to management literature. Firstly, we contribute to the advancement of trust research by theoretically distinguishing and empirically examining the concepts of feeling trusted and trusting others as parallel yet distinct outcomes of digital work environments. Previous research has conceptualized trust as a unidimensional construct or has focused exclusively on interpersonal trust. Our study demonstrates that these two dimensions respond to different facets of digital tool usage. Specifically, feelings of being trusted appear to be more sensitive to signals of organizational empowerment and autonomy, while trust in others is more strongly influenced by improvements in communication quality and interpersonal connection. This distinction is significant because organizations that seek to establish trust through technology investments must comprehend which trust dimension their initiatives are likely to impact. Secondly, we seek to reconcile the competing theoretical perspectives on the role of technology in shaping workplace relationships. Extant literature has oscillated between optimistic accounts emphasizing transparency and connectivity, and pessimistic warnings about surveillance and algorithmic control. Rather than evaluating these perspectives against each other, the focus is directed towards identifying the specific mechanisms and conditions under which each perspective is valid. By demonstrating that communication quality and psychological safety mediate the positive effects of digital tool usage, while perceived surveillance and technostress suppress these benefits, a more nuanced understanding of when and how digitalization builds or erodes trust is provided. This conditional perspective sheds light on the discordant findings in extant research and establishes a more pragmatic foundation for both theory and practice. Thirdly, we propose an expansion of the conceptualization of digital work environments beyond conventional communication platforms. While most research has centered on email, instant messaging, or video conferencing, contemporary employees are increasingly engaging with immersive technologies such as virtual reality training systems and ambient monitoring through Internet of Things devices. The incorporation of advanced technologies into the measurement of digital tool usage intensity enables the comprehensive assessment of the digital experiences that collectively influence contemporary work environments. This comprehensive approach yields insights that persist in their relevance as organizations continue to adopt emerging technologies, ensuring that our findings address the future of work rather than merely documenting current practices.

## 2 Literature review and hypotheses

To understand how digital tool usage intensity shapes the reciprocal dynamics of trust, we draw upon Signaling Theory and Social Exchange Theory (SET). Signaling theory suggests that organizational resource allocation serves as a symbolic message about how the organization values its employees (Connelly et al., 2011). Simultaneously, SET posits that workplace relationships are governed by the norm of reciprocity (Kahan, 2002). We posit that digital tool usage, ranging from collaborative platforms to immersive VR and IoT interfaces, acts as a salient environmental signal that triggers cognitive and affective processing (communication quality and psychological safety), ultimately influencing both the employee’s perception of being trusted and their willingness to trust others.

### 2.1 Digital tool usage and the trust dynamics

Past research has long debated the role of technology in relational signaling. We posit that high-intensity usage of advanced digital tools signals organizational investment in the employee’s capability and autonomy. Unlike basic prescriptive tools, high-embeddedness digital tools often require user discretion and convey a message of insider status (Angwin et al., 2009). When an organization empowers an employee with sophisticated digital infrastructure, it effectively signals: “We trust you to handle critical information and leverage complex resources.” Consequently, employees interpret this digital enablement as a manifestation of the organization’s reliance on their professional judgment.

*H1a*: Digital tool usage intensity is positively associated with employees’ feelings trusted by their supervisors and the organization.

Digital Tool Usage Intensity and Trusting Others Furthermore, digital tool usage creates a structural foundation for trusting others. Digital tools, particularly collaborative and social platforms, increase the frequency and transparency of interactions. According to the Contact Hypothesis, increased interaction, facilitated here by digital means, reduces uncertainty and fosters familiarity (Lowe, 2021; Muskat et al., 2022). When employees frequently utilize digital tools to coordinate with supervisors and colleagues, the predictability of workflows increases. If the digital infrastructure functions reliably as a support system, employees are likely to extend this trust to the providers of the system (the organization) and the counterparts they interact with supervisors or colleagues (Avtalion et al., 2024).

*H1b*: Digital tool usage intensity is positively associated with employees’ trust in their supervisors, colleagues, and the organization.

### 2.2 The mediating mechanisms: unpacking the black box

We propose that the link between digital tool usage and trust is transmitted through enhanced communication quality. High-intensity digital tool usage, especially through immersive media such as VR, can heighten telepresence and social presence, the experience of “being there” and “being with others”, thereby improving the richness of digitally mediated interaction (Steuer, 1992; Biocca et al., 2003; Cummings and Bailenson, 2016). Digital tools reduce information asymmetry by providing real-time access to data and fostering transparent dialogue (Aben et al., 2021). When communication quality is high, ambiguity decreases. From the perspective of the individual employee, the seamless transmission of information is indicative of organizational transparency, thereby fostering a culture of trust (enhancing Trusting Others). The capacity to access information unencumbered by bureaucratic gatekeeping signifies an organizational paradigm shift, where employees are regarded as autonomous and reliable entities (enhancing Feeling Trusted).

*H2*: Communication quality mediates the relationship between digital tool usage and (a) feeling trusted and (b) trusting others.

Psychological safety also serves as a critical affective mechanism. The increased use of digital tools has been shown to positively impact employee empowerment by augmenting their capabilities. IoT-enabled systems can expand employees’ access to real-time monitoring, analytics, and information sharing, thereby improving decision support and coordination when implemented as enabling rather than coercive infrastructures (Lee and Lee, 2015; Westergren et al., 2024). When digital tools are designed to facilitate rather than dictate work processes, they can foster a sense of structural empowerment among users (Parker and Grote, 2022). This empowerment fosters psychological safety, defined as the belief that the work environment is conducive to interpersonal risk-taking. Employees who feel empowered by technology perceive this autonomy as a strong signal of trust from the leader (Feeling Trusted). Conversely, this safety fosters an environment conducive to employee vulnerability and reliance on the organization, thereby completing the social exchange loop.

*H3*: Psychological safety mediates the relationship between digital tool usage and (a) feeling trusted and (b) trusting others.

### 2.3 The moderating effects: the dark side of digitization

While the baseline logic suggests a positive impact, the Digital Paradox implies that the interpretation of digital signals is context dependent. We introduce two critical boundary conditions: Perceived Digital Surveillance and Technostress.

The moderating role of perceived digital surveillance refers to the extent to which employees feel their behavior is scrutinized by digital systems. Classic work on the information panopticon and workplace surveillance suggests that digital systems translate activities into traceable information and centralize oversight, even when discretion appears to be delegated (Zuboff, 1988; Sewell and Wilkinson, 1992). More recent work on algorithmic management shows that digital infrastructures can direct, evaluate, and discipline workers through recording, rating, recommendation, and matching systems (Kellogg et al., 2020; Möhlmann et al., 2021). The present study posits that surveillance has the capacity to modify the attribution of digital tool usage (Wang and Tucker, 2021; Mettler, 2024). In situations where individuals perceive an elevated degree of surveillance, the empowering potential of digital tools is often distorted, leading to their utilization as instruments of control rather than empowerment (Kensbock and Stöckmann, 2021). Rather than perceiving the provision of digital tools as a resource for autonomy, employees interpret them as electronic leashes, a phenomenon often referred to as the Panopticon effect (Leclercq-Vandelannoitte, 2023). Despite the technically proficient communication, the underlying intent is met with suspicion. This attribution shift weakens the positive effect of digital tool usage on psychological safety and feeling trusted. In essence, if there is a perception of being observed, a sense of distrust is instigated, and the observer is no longer regarded with confidence, irrespective of the sophistication of the surveillance apparatus.

*H4*: Perceived digital surveillance negatively moderates the indirect effect of digital tool usage intensity on trust outcomes. Specifically, the positive effects mediated by psychological safety are weaker when perceived surveillance is high.

The moderating role of technostress, drawing on the Conservation of Resources (COR) theory, is examined in this study. Technostress, defined as stress caused by the inability to cope with new technologies in a healthy manner, has been shown to deplete employees’ cognitive resources (Rohwer et al., 2022). The phenomenon of technostress, characterized by the accumulation of stressors related to technology, has been demonstrated to induce a state of cognitive overload and anxiety (Pang and Wang, 2025). In situations where employees are inundated with constant connectivity, system updates, or demands for multitasking, they often find themselves with limited psychological resources to fully appreciate the “empowerment” or “communication” benefits that digital tools are designed to provide (Lin, 2025). Consequently, these instruments, which were initially conceived as facilitators of support, have become a source of threat. This negative affective state has been shown to impede the translation of digital tool usage into positive communication quality and psychological safety, thereby severing the link to trust.

*H5*: Technostress negatively moderates the relationship between digital tool usage intensity and the mediating mechanisms (communication quality and psychological safety), such that the positive indirect effects on trust outcomes are attenuated when technostress is high.

## 3 Methodology

### 3.1 Participants and procedures

To empirically test our hypotheses, we employed a survey-based methodology. Given that our research focuses on subjective psychological states and behavioral responses—specifically digital tool usage, perceived trust, and feeling trusted—self-reported data from employees was deemed the most appropriate approach. We utilized a purposive sampling strategy, recruiting full-time employees through university alumni networks. We specifically targeted industries characterized by high degrees of digitization and strong demands for innovation and collaboration, such as internet services, software development, online operations, and digital platform management. To ensure data quality and sample representativeness, participation was restricted to full-time employees with a tenure of at least 6 months in their current organization, ensuring they possessed sufficient knowledge of the firm’s digital tools and trust climate.

Because our model relies on self-reported data for all key variables (e.g., digital tool usage, communication quality, surveillance, and trust constructs), reliance on single-source, cross-sectional data could introduce common method bias (CMB). To mitigate this risk, we adopted a two-wave time-lagged design to create temporal separation between the measurement of independent/moderating variables and dependent/mediating variables.

Time 1 (T1): Participants reported on their digital tool usage, perceived communication quality, perceived digital surveillance, technostress, and demographic information.

Time 2 (T2): Approximately 4 weeks later, the same participants reported on their psychological safety, feeling trusted, and trust in others.

Prior to the main survey, a pilot study was conducted with a small sample of the target population to ensure item clarity and readability; minor wording adjustments were made based on feedback. In the first phase (T1), we distributed 500 questionnaires. After excluding invalid responses, defined as those with significant missing data, completion times below a reasonable threshold, or failure to pass attention checks (e.g., logic trap questions), we obtained 472 valid responses. Four weeks later, we distributed the T2 survey to these valid T1 respondents. We received 433 responses. We further cleaned the data by removing participants who could not be successfully matched to their T1 data, as well as those exhibiting response patterns indicative of insufficient effort (e.g., “straight lining” or selecting the same option consecutively). The final dataset consisted of 428 valid matched responses, representing an effective retention rate of approximately 85.6%.

The demographic profile of the final sample is well-aligned with the research context. Males constituted slightly more than half of the sample. The average age was approximately 30 years. Most participants held a bachelor’s degree or higher and were employed in digitization sectors (e.g., internet services and software development). Collectively, these procedural remedies, including temporal separation, anonymized matching codes, and the use of attention checks, effectively minimized the potential influence of common method bias on our findings.

### 3.2 Questionnaire development

All core constructs in this study were measured using well-established scales that have been widely applied in prior research and validated in the Chinese organizational context. To ensure linguistic equivalence and cultural appropriateness, we followed a rigorous translation-back-translation procedure. Two researchers with overseas academic experience first translated the original English scales into Chinese. Subsequently, a bilingual researcher who had not been exposed to the original scales performed back-translation into English. Any items with ambiguous meanings were carefully refined through iterative discussion among the translation team to ensure semantic precision and cultural relevance.

Unless otherwise specified, all items were assessed using a five-point Likert scale, where 1 represented “strongly disagree” and 5 represented “strongly agree.” Higher scores indicated greater intensity or stronger perceptions of the respective construct.

#### 3.2.1 Digital tool usage

Digital tool usage intensity was measured using a scale adapted from established research on information and communication technology (ICT) usage in organizational settings (Carlson and Zmud, 1999). The scale assesses the frequency and depth with which employees utilize various digital work tools in their daily operations, including instant messaging platforms, online collaboration systems, project management software, video conferencing applications, and mobile office tools.

The scale comprises seven items covering multiple dimensions of digital tool engagement, such as usage frequency, functional breadth, and task dependency. Following minor semantic refinements based on pilot testing results, the scale demonstrated strong internal consistency in the formal sample, with a Cronbach’s alpha coefficient of 0.877.

#### 3.2.2 Digital communication quality

Digital communication quality was measured using a scale adapted from established research on social presence and media richness (Carlson and Zmud, 1999). The scale captures employees’ overall perceptions of information adequacy, interaction fluency, and emotional expression clarity when communicating with colleagues and supervisors through digital tools.

The scale consists of four items designed to assess the effectiveness of digitally mediated workplace communication. The scale demonstrated adequate internal consistency in the current sample, with a Cronbach’s alpha coefficient of 0.832.

#### 3.2.3 Digital monitoring perception

Employees’ subjective perceptions of digital monitoring were assessed using a scale adapted from research on electronic performance monitoring (EPM) in organizational settings (Stanton, 2000; Jeske and Santuzzi, 2015). The scale comprises three items that focus on employees’ awareness of systematic behavior tracking, work process recording, and real-time performance data visualization within digital systems. This measure captures the extent to which employees perceive themselves as being subject to digital surveillance and data collection in their daily work activities. The scale demonstrated acceptable internal consistency in the current sample, with a Cronbach’s alpha coefficient of 0.753.

#### 3.2.4 Technostress

Technostress was measured using a simplified adaptation of items from the technostress creators scale developed by Tarafdar et al. (2007) and Ragu-Nathan et al. (2008). The scale specifically draws on dimensions related to technology overload, technology complexity, and technology uncertainty to assess the burden and strain employees’ experience from frequent use of digital tools in their work.

The scale consists of three items designed to capture the psychological tension arising from digitally mediated work demands. This measure reflects the extent to which employees perceive digital technologies as sources of pressure and cognitive strain rather than facilitators of productivity. The scale demonstrated acceptable internal consistency in the current sample, with a Cronbach’s alpha coefficient of 0.740.

#### 3.2.5 Psychological safety

Psychological safety was measured using a contextualized adaptation of Edmondson's (1999) team psychological safety scale. The original scale assesses the extent to which employees feel safe to express their views, voice questions, or acknowledge mistakes within their team environment. Given that this study focuses on individual employee experiences within digitally mediated communication contexts, we modified references to “team” and “meetings” in the original items to reflect the specific nature of digital workplace interactions.

The adapted scale comprises four items that capture employees’ perceptions of interpersonal risk and openness in their work environment. This measure reflects the degree to which employees believe they can engage in candid communication and take interpersonal risks without fear of embarrassment or retribution, particularly in technology-mediated settings. The scale demonstrated strong internal consistency in the current sample, with a Cronbach’s alpha coefficient of 0.830.

#### 3.2.6 Feeling trusted

Employee perceptions of being trusted were assessed using a scale adapted from Lau et al. (2014) and Hao et al. (2021) felt trust inventory and subsequent operationalizations of the felt reliance and felt disclosure dimensions in later research (Zheng et al., 2019). The scale focuses on employees’ subjective experiences of trust extended by their immediate supervisors.

The scale consists of five items designed to measure the extent to which employees perceive that their supervisors place confidence in their capabilities and discretion. These items collectively reflect employees’ perceptions of being viewed as dependable and trustworthy by their organizational superiors, which represents a distinct psychological experience from employees’ own trust in others. The scale demonstrated strong internal consistency in the formal sample, with a Cronbach’s alpha coefficient of 0.866.

#### 3.2.7 Trusting others

Employee trust in others was measured using an integrated scale that draws from established research on interpersonal trust and organizational trust in workplace settings (Mayer et al., 1995; McAllister, 1995). The scale was designed to capture both cognitive trust, which reflects assessments of others’ competence and reliability, and affective trust, which encompasses perceptions of emotional support and benevolence. This dual conceptualization allows for a comprehensive assessment of employees’ trust toward colleagues, teams, and the broader organization.

The scale comprises five items that collectively assess the extent to which employees believe others in their work environment are dependable and well-intentioned. These items reflect employees’ willingness to be vulnerable in their organizational relationships based on positive expectations regarding others’ intentions and behaviors. The scale demonstrated strong internal consistency in the current sample, with a Cronbach’s alpha coefficient of 0.875.

#### 3.2.8 Control variables

Drawing on prior research examining digital work environments and trust perceptions, this study controlled several individual characteristics that may influence employees’ trust-related perceptions and behaviors. The control variables included employee gender, age, organizational tenure, and current work arrangement (on-site or remote or hybrid work).

All control variables were measured through single-item self-report questions. This comprehensive set of controls allows for more precise estimation of the relationships between digital tool usage, mediating mechanisms, and trust outcomes by accounting for individual differences that might otherwise confound the relationships we focus on.

### 3.3 Data analysis

#### 3.3.1 Confirmatory factor analysis and common method bias tests

To assess the distinctiveness of the focal constructs, we conducted a series of confirmatory factor analyses (CFA) using the SEM module in Stata. The hypothesized seven-factor model, including digital tool use, digital communication quality, perceived digital monitoring, technostress, psychological safety, feeling trusted, and trusting others, demonstrated a good fit to the data (*χ*^2^/df = 1.076, CFI = 0.995, TLI = 0.994, RMSEA = 0.013, SEMR = 0.032; see Table 1). This model fit the data better than several alternative models in which theoretically related constructs were combined (e.g., merging feeling trusted and trusting others into a single trust factor or merging monitoring and technostress into a single digital strain factor). These results indicate that the focal variables in this study possess satisfactory discriminant validity and represent empirically distinct constructs.

**Table 1:** Validity analysis (*N* = 428).

Models		$\chi^{2}/\mathrm{df}$	CFI	TLI	RMSEA	SRMR
Seven-factor model	DIGUSE; COMM; MON; TS; PSY; FT; TR	1.076	0.995	0.994	0.013	0.032
Six-factor model	DIGUSE; COMM; MON; TS; PSY; FT + TR	3.112	0.844	0.827	0.070	0.085
Five-factor model	DIGUSE; COMM; MON + TS; PSY; FT + TR	3.748	0.795	0.775	0.080	0.096
One-factor model	DIGUSE + COMM + MON + TS + PSY + FT + TR	8.620	0.419	0.377	0.134	0.160

Because all focal variables were measured via self-reported questionnaires, common method bias (CMB) may threaten the validity of the findings. To address this concern, we first performed Harman’s single-factor test. An exploratory factor analysis including all measurement items extracted 6 factors with eigenvalues greater than 1, and the largest factor accounted for only 18.25% of the total variance, which is well below the commonly used 40% threshold. This suggests that no single factor dominated the covariance among the measures. Furthermore, to move beyond the limitations of Harman’s single-factor test, we implemented a more rigorous Common Latent Factor (CLF) approach. We introduced an unmeasured latent method construct into our seven-factor CFA model and allowed it to load on all observed items. The results showed that the inclusion of the CLF did not significantly improve the model fit ( $\mathrm{CFI}={\mathrm{CFI}}_{\mathrm{CLF}}-{\mathrm{CFI}}_{\text{Baseline}}<0.05$ ).

#### 3.3.2 Descriptive statistics and correlation analysis of variables

Table 2 presents the means, standard deviations, and Pearson correlations among the study variables. Digital tool usage (DTU) exhibits significant and positive correlations with both mediating mechanisms: communication quality (DCQ) (*r* = 0.497, *p* < 0.001) and psychological safety (PSYS) (*r* = 0.447, *p* < 0.001). Furthermore, DTU is positively and significantly associated with the two core outcome variables: feeling trusted (FT) (*r* = 0.427, *p* < 0.001) and trusting others (TRO) (*r* = 0.342, *p* < 0.001). Regarding the interrelationships among mediators and outcomes, DCQ is positively associated with FT (*r* = 0.268, *p* < 0.001), and TRO (*r* = 0.485, *p* < 0.001). PSYS demonstrates a strong positive correlation with FT (*r* = 0.707, *p* < 0.001) and a moderate positive correlation with TRO (*r* = 0.444, *p* < 0.001), suggesting a close theoretical linkage between safety and trust perceptions. In terms of the “dark side” variables, DTU is also positively correlated with perceived monitoring (PM) (*r* = 0.392, *p* < 0.001) and technostress (TECS) (*r* = 0.302, *p* < 0.001). PM, in turn, shows a significant negative correlation with FT (*r* = −0.119, *p* < 0.05) and TRO (*r* = −0.138, *p* < 0.01). TECS also shows negative correlations with FT (*r* = −0.141, *p* < 0.01) and TRO (*r* = −0.120, *p* < 0.05). Overall, these correlations provide preliminary support for our dual-pathway framework, where digital tools simultaneously trigger empowerment signals (via communication quality and psychological safety) and control signals (via perceived monitoring and technostress).

**Table 2:** Descriptive statistics and correlation analysis.

	DTU	DCQ	PM	TECS	PSYS	FT	TRO
DTU	1.000						
DCQ	0.497***	1.000					
PM	0.392***	−0.243***	1.000				
TECS	0.302***	−0.133**	0.147**	1.000			
PSYS	0.447***	0.438***	−0.143**	−0.104*	1.000		
FT	0.427***	0.268***	−0.119*	−0.141**	0.707***	1.000	
TRO	0.342***	0.485***	−0.138**	−0.120*	0.444***	0.322***	1.000
Mean	2.983	2.957	3.019	2.926	3.114	3.543	2.963
SD	0.576	0.613	0.628	0.597	0.821	0.656	0.589

*N* = 428, ***p < 0.001, **p < 0.01, *p < 0.05.

#### 3.3.3 Hypothesis testing

Prior to hypothesis testing, we assessed the potential threat of multicollinearity given the moderate correlations among predictors (e.g., *r* = 0.49 between digital tool usage and communication quality). The results are shown in Table 3. As all VIF values were well below the stringent threshold of 3.0, multicollinearity does not pose a threat to our regression estimates.

**Table 3:** VIF results.

Variable	VIF	1/VIF
digital_use	1.61	0.620185
comm_quality	1.60	0.623983
Monitoring	1.38	0.723876
psy_safety	1.35	0.741949
tech_stress	1.11	0.901333
remote_mode	1.06	0.942688
Tenure	1.04	0.960296
Position	1.03	0.972765
Gender	1.03	0.975058
age_group	1.02	0.983511
Mean VIF	1.22	

*Main Effects*: Table 4 presents the regression results for the main effects of digital tool usage intensity on trust outcomes. After controlling for demographic and job-related variables, digital tool usage was found to be positively and significantly associated with feeling trusted (*b* = 0.4680, *p* < 0.001, Model 2) and trusting others (*b* = 0.3407, *p* < 0.001, Model 4). These findings provide strong empirical support for Hypothesis 1a and Hypothesis 1b, suggesting that higher engagement with digital tools serves as a significant signal fostering mutual trust in the workplace.

**Table 4:** Baseline results.

	(1)	(2)	(3)	(4)
	feeling_trusted	feeling_trusted	trusting_others	trusting_others
digital_use	0.4866*** (9.7470)	0.4680*** (9.2956)	0.3495*** (7.5085)	0.3407*** (7.1899)
Gender		−0.0391 (−0.6787)		−0.0274 (−0.5056)
age_group		0.0319 (1.2547)		−0.0003 (−0.0116)
Position		0.0636* (2.4589)		0.0434 (1.7826)
Tenure		0.0144* (2.1173)		0.0063 (0.9818)
remote_mode		0.0928 (1.5134)		0.0421 (0.7283)
Constant	2.0919*** (13.7922)	1.7817*** (9.4594)	1.9206*** (13.5835)	1.7868*** (10.0789)
*N*	428	428	428	428
Adjusted-*R*^2^	0.1804	0.1950	0.1148	0.1137

*N* = 428, ***p < 0.001, **p < 0.01, *p < 0.05.

*Mediation Effects*: We examined the mediating roles of communication quality and psychological safety (H2 and H3). As shown in Table 5, digital tool usage was positively related to communication quality (*b* = 0.51, *p* < 0.001, Model 1). Communication quality was positively associated with both feeling trusted (*b* = 0.27, *p* < 0.001, Model 2) and trusting others (*b* = 0.46, *p* < 0.001, Model 3). Similarly, Table 6 indicates that digital tool usage significantly enhanced psychological safety (*b* = 0.61, *p* < 0.001, Model 1). Subsequently, psychological safety positively predicted feeling trusted (*b* = 0.56, *p* < 0.001, Model 2) and trusting others (*b* = 0.31, *p* < 0.001, Model 3).

**Table 5:** Mediation results: communication quality.

	(1)	(2)	(3)
	comm_quality	feeling_trusted	trusting_others
digital_use	0.5106*** (11.2307)		
Gender	−0.0341 (−0.6562)	−0.0275 (−0.4491)	−0.0108 (−0.2139)
age_group	−0.0193 (−0.8433)	0.0327 (1.2090)	0.0073 (0.3296)
Position	−0.0103 (−0.4400)	0.0687* (2.5020)	0.0489* (2.1615)
Tenure	0.0023 (0.3707)	0.0179* (2.4781)	0.0065 (1.0948)
remote_mode	0.1270* (2.2930)	0.1227 (1.8779)	0.0030 (0.0561)
comm_quality		0.2690*** (5.3483)	0.4649*** (11.2211)
Constant	1.4657*** (8.6178)	2.3247*** (11.8448)	1.3984*** (8.6504)
*N*	428	428	428
Adjusted-*R*^2^	0.2475	0.0915	0.2340

*N* = 428, ***p < 0.001, **p < 0.01, *p < 0.05.

**Table 6:** Mediation results: psychological safety.

	(1)	(2)	(3)
	psy_safety	feeling_trusted	trusting_others
digital_use	0.6067*** (9.7475)		
Gender	0.0248 (0.3489)	−0.0520 (−1.1438)	−0.0341 (−0.6586)
age_group	0.0353 (1.1242)	0.0104 (0.5211)	−0.0134 (−0.5859)
Position	0.0550 (1.7219)	0.0338 (1.6521)	0.0271 (1.1630)
Tenure	0.0234** (2.7798)	0.0030 (0.5473)	0.0008 (0.1254)
remote_mode	0.1342 (1.7697)	0.0431 (0.8921)	0.0288 (0.5231)
psy_safety		0.5577*** (19.8475)	0.3147*** (9.8258)
Constant	0.8370*** (3.5942)	1.6825*** (13.9391)	1.9479*** (14.1568)
*N*	428	428	428
Adjusted-*R*^2^	0.2145	0.4988	0.1905

*N* = 428, ***p < 0.001, **p < 0.01, *p < 0.05.

**Figure 1:**
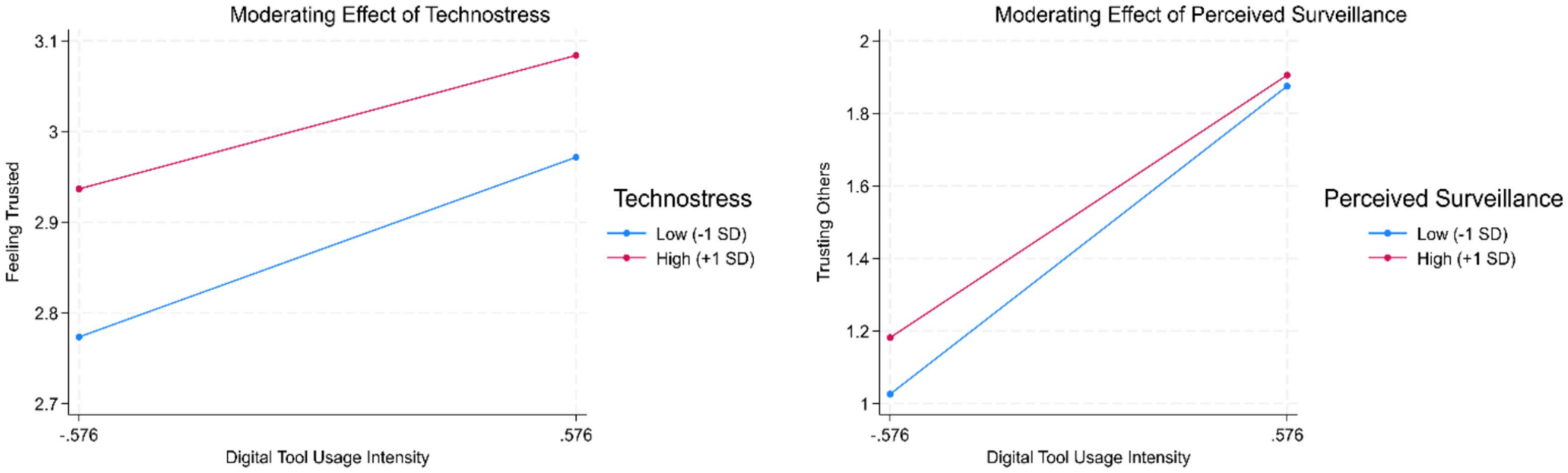
Moderation effects.

*Moderation Effects*: H4 and H5 assumed that the positive effects of digital tool usage would be attenuated by perceived digital surveillance and technostress. Table 7 reports the moderation analysis results. First, regarding surveillance (H4), the interaction term between digital tool usage and perceived monitoring was negative and significant for both trusting others (*b* = −0.05, *p* < 0.001, Model 1) and feeling trusted (*b* = −0.03, *p* < 0.01, Model 2). This indicates that the trust-building effect of digital tools is weaker when employees perceive high levels of surveillance. Second, regarding technostress (H5), the interaction term between digital tool usage and technostress was also negative and significant for trusting others (*b* = −0.06, *p* < 0.001, Model 3) and feeling trusted (*b* = −0.10, *p* < 0.001, Model 4). This suggests that the benefits of digital tools are diminished when employees experience high technology-induced stress. The results are also shown in Figure 1.

**Table 7:** Moderation results.

	(1)	(2)	(3)	(4)
	trusting_others	feeling_trusted	trusting_others	feeling_trusted
digital_use	0.4727*** (9.4066)	0.6484*** (12.3875)	0.3216*** (6.6670)	0.4407*** (9.1721)
digital_use* monitoring	−0.0536*** (−5.1398)	−0.0300** (−2.7661)		
digital_use* tech_stress			−0.0563*** (−5.3302)	−0.0998*** (−9.4925)
Monitoring	−0.0342 (−0.6400)	−0.2588*** (−4.6451)		
tech_stress			−0.1526** (−2.9222)	−0.2495*** (−4.7985)
Gender	−0.0191 (−0.3690)	−0.0336 (−0.6229)	−0.0269 (−0.5122)	−0.0376 (−0.7169)
age_group	−0.0078 (−0.3412)	0.0294 (1.2333)	−0.0165 (−0.7056)	0.0032 (0.1364)
Position	0.0365 (1.5736)	0.0566* (2.3415)	0.0290 (1.2220)	0.0380 (1.6049)
Tenure	0.0005 (0.0822)	0.0080 (1.2482)	0.0032 (0.5077)	0.0089 (1.4371)
remote_mode	0.0323 (0.5868)	0.0740 (1.2886)	0.0633 (1.1269)	0.1297* (2.3193)
Constant	2.0132*** (10.6551)	2.3464*** (11.9224)	1.9059*** (9.7075)	2.0346*** (10.4031)
*N*	428	428	428	428
Adjusted-*R*^2^	0.1940	0.2963	0.1663	0.3344

*N* = 428, ***p < 0.001, **p < 0.01, *p < 0.05.

#### 3.3.4 Endogeneity and robustness checks

To address potential endogeneity and simultaneity bias arising from the simultaneous collection, we performed the Durbin–Wu–Hausman (DWH) test using an instrumental variable (IV) approach. Specifically, we employed the “leave-one-out peer average” as an instrument, calculated as the average digital tool usage intensity of all other respondents within the same industry sector (excluding the focal respondent *i*).

Theoretically, this instrument satisfies the two requisite conditions for a valid IV. First, industry-level digital norms strongly drive an individual employee’s tool usage frequency (relevance). Second, the average digital behavior of peers in the industry is unlikely to directly influence a focal employee’s subjective psychological states, such as technostress or perceived surveillance, except through the employee’s own usage patterns (exclusion restriction). We conducted Two-Stage Least Squares (2SLS) regressions followed by the estat endogenous test in Stata. The Wu–Hausman *F*-test failed to reject the null hypothesis of exogeneity (feeling_trusted: *F* = 0.4689, *p* = 0.4938; trusting_others: *F* = 0.2541, *p* = 0.6145). These results indicate that the independent variable can be treated as exogenous, supporting the consistency and efficiency of our original structural estimates (Table 8).

**Table 8:** IV results.

	(1)	(2)
	feeling_trusted	trusting_others
digital_use	0.8680*** (7.2956)	0.6340*** (4.5310)
Gender	−0.0352 (−0.7163)	−0.0371 (−0.1051)
age_group	−0.0034 (−0.1523)	−0.0007 (−0.0301)
Position	0.1245* (2.1667)	0.0522 (1.1817)
Tenure	0.0574 (0.9263)	0.0073 (1.0504)
remote_mode	0.0569 (1.2541)	0.0591 (0.8353)
Constant	3.0396*** (12.1788)	2.0898*** (7.0789)
*N*	428	428
Adjusted-*R*^2^	0.2103	0.1409
Wu–Hausman	0.4689 (*p* = 0.4938)	0.2541 (*p* = 0.6145)

*N* = 428, ***p < 0.001, **p < 0.01, *p < 0.05.

## 4 Discussions and conclusion

### 4.1 Discussions of findings

This study examined the mechanisms through which digital tool usage influences workplace trust dynamics. The findings of this study provide substantial support for an integrated signaling-social exchange framework, while concurrently revealing that digitalization functions as a double-edged sword, with its effects contingent upon implementation choices and employee experiences.

In accordance with Hypotheses 1a and 1b, the intensity of digital tool usage had a positive influence on feelings of being trusted and the propensity to trust others. This finding resolves a fundamental tension in the literature between optimistic accounts emphasizing technology-enabled transparency and pessimistic warnings about digital surveillance (Zuboff, 1988; Sewell and Wilkinson, 1992; Kellogg et al., 2020; Leonardi, 2020). The findings of this study indicate that, at baseline levels of monitoring and stress, organizational investment in sophisticated digital infrastructure is indicative of confidence in employee capabilities. Additionally, increased interaction frequency has been shown to reduce uncertainty regarding others’ intentions. The stronger effect on feeling trusted indicates that employees interpret digital resource provision as particularly salient evidence of organizational reliance on their judgment. This lends support to signaling theory’s premise that resource allocation decisions communicate symbolic messages about employment relationships (Connelly et al., 2011).

Our dual mediation findings represent the study’s central theoretical contribution. Communication quality and psychological safety operate through distinct mechanisms that differentially influence the two trust dimensions. Communication quality showed stronger associations with trusting others, reflecting an informational logic whereby digital tools reduce uncertainty through enhanced transparency and interaction fluency. This aligns with cognitive trust research emphasizing systematic assessment of others’ reliability (Mayer et al., 1995). Psychological safety, conversely, demonstrated stronger effects on feeling trusted, suggesting that when digital tools support rather than constrain autonomy, employees interpret this as evidence of organizational confidence. This pattern reveals that trust formation in digital environments requires both informational adequacy and relational safety.

The moderation analyses confirmed Hypotheses 4 and 5, validating the digital paradox concept. Perceived surveillance and technostress significantly attenuated the positive effects of digital tool usage. Surveillance triggered what we term signal reinterpretation, whereby identical technologies conveyed control rather than empowerment when employees perceived extensive behavior tracking. This finding extends surveillance research by demonstrating that monitoring perceptions fundamentally alter the meaning employees extract from technology investments rather than merely adding negative effects (Wang and Tucker, 2021). Technostress operated through resource depletion, whereby cognitive burdens from system complexity and connectivity demands left employees unable to process communication improvements or safety signals effectively. These boundary conditions explain inconsistent findings in prior digitalization research and demonstrate that implementation of choices regarding monitoring intensity and system design critically determine whether digital tools build or erode trust.

A synthesis of the findings reveals that digitalization exerts trust effects that are bidirectional, asymmetric, and conditional. The dual-pathway model elucidates that the comprehensive establishment of trust necessitates the consideration of two distinct mechanisms: informational transparency and relational safety. These mechanisms function through disparate psychological processes. The findings concerning boundary conditions indicate that positive potential can be reversed when implementations trigger control attributions or resource depletion. These results suggest that technology investments convey significant symbolic messages concerning organizational philosophy. It is imperative for leaders to acknowledge that the monitoring of intensity, system complexity, and autonomy preservation constitute strategic decisions that delineate whether digitalization signals trust or control. These decisions are not merely technical implementation details.

### 4.2 Limitations and future research directions

First, although we employed a two-wave time-lagged design to minimize common method bias, the data remains correlational, which precludes definitive causal inferences. For instance, high levels of pre-existing trust might lead supervisors to grant employees access to more advanced digital tools. Future research should employ longitudinal designs with multiple time points or field experiments to rigorously establish the causal impact of digital tool intensity on trust development.

Second, all variables in this study were assessed using self-reported measures. While this is appropriate for capturing subjective psychological states (e.g., feeling trusted and psychological safety), reliance on self-reports for digital tool usage may not perfectly reflect actual behavior. Future studies would benefit from incorporating objective data, such as system logs or screen time analytics, and adopting multi-source designs that include supervisor ratings of trust to enhance measurement validity.

Third, the purposive nature of our sampling strategy may have inadvertently constrained the natural variance of our dataset. To ensure respondents possessed sufficient exposure to advanced digital tools, our sample was heavily concentrated within core technical roles in highly digitized sectors. While this controlled for unobserved heterogeneity related to basic digital literacy, it resulted in a highly homogeneous sample of “digital natives.” Consequently, the employees in our study exhibited uniform cognitive schemas and tolerance thresholds regarding technostress and digital surveillance, leading to range restriction in our variance–covariance matrix. Future research must extend this framework to more diverse, cross-functional organizational settings to introduce natural organizational noise and test the external validity and robustness of our dual-pathway model across heterogeneous populations.

## Data Availability

The raw data supporting the conclusions of this article will not be made publicly available due to the sensitive nature of the survey questions regarding workplace dynamics and the need to protect participant privacy and anonymity. The informed consent signed by the participants did not include permission for public data sharing. However, anonymized data may be available from the corresponding author upon reasonable request. Requests to access the datasets should be directed to yanze517@163.com.
