# Supplementary material for: Reshaping relational social capital in the digital age: how digital tool usage influences the dual trust outcomes
Source: Front Psychol. 2026 May 15;17:1759562. doi: 10.3389/fpsyg.2026.1759562 (PMC13218974; doi:10.3389/fpsyg.2026.1759562)
Supplement: Supplementary file 1 [file Supplementary_file_1.pdf]

**Item**	**Technological Modality / Tool Category**	**Standardized Loading**	**z-value**	**p-value**
DU1	Instant messaging & team communication	0.728	27.72	***
DU2	Online collaborative document systems	0.740	29.03	***
DU3	Project & task management platforms	0.721	27.01	***
DU4	**Virtual reality (VR) / AR interfaces**	0.704	25.30	***
DU5	**IoT-based operational dashboards**	0.713	26.19	***
DU6	Algorithm-driven task allocation systems	0.765	30.19	***
DU7	Mobile office (attendance, approvals)	0.782	32.14	***
